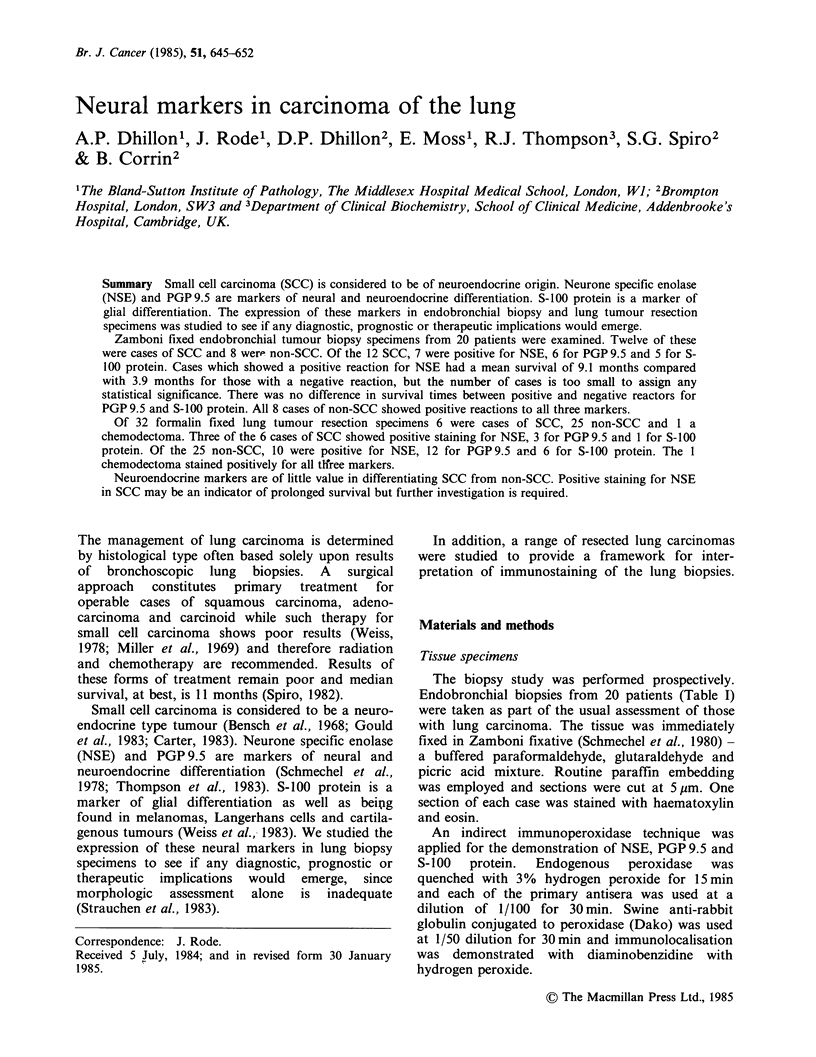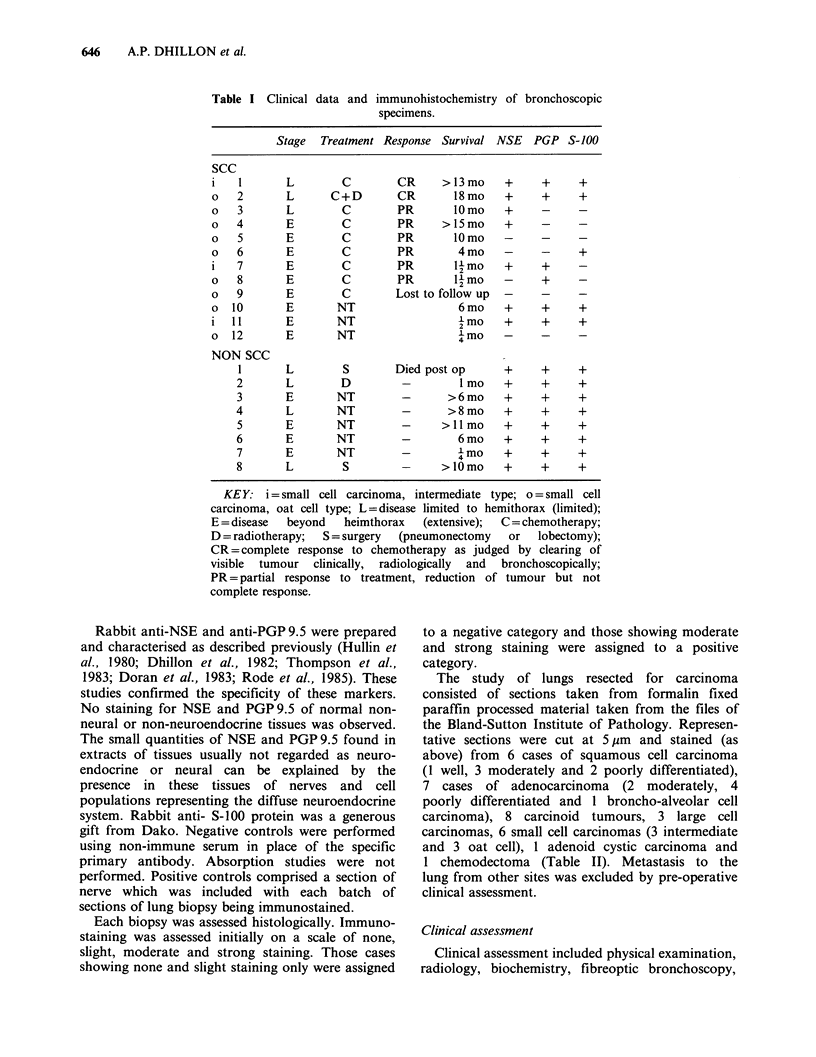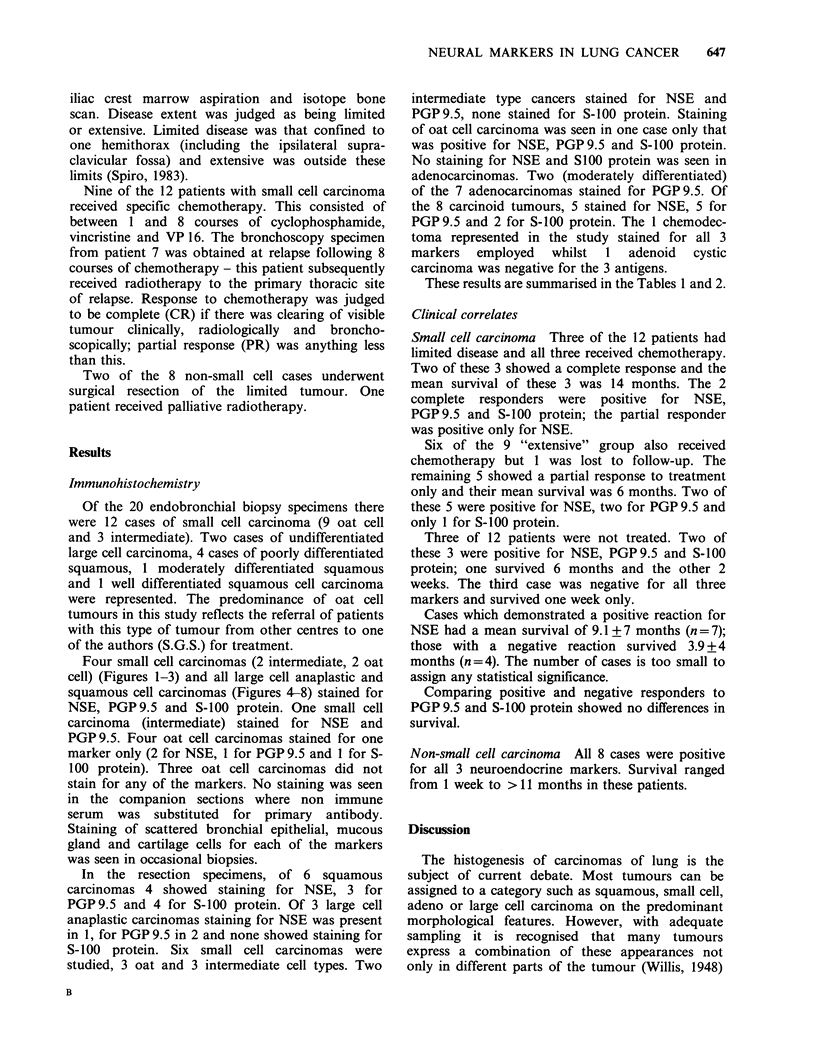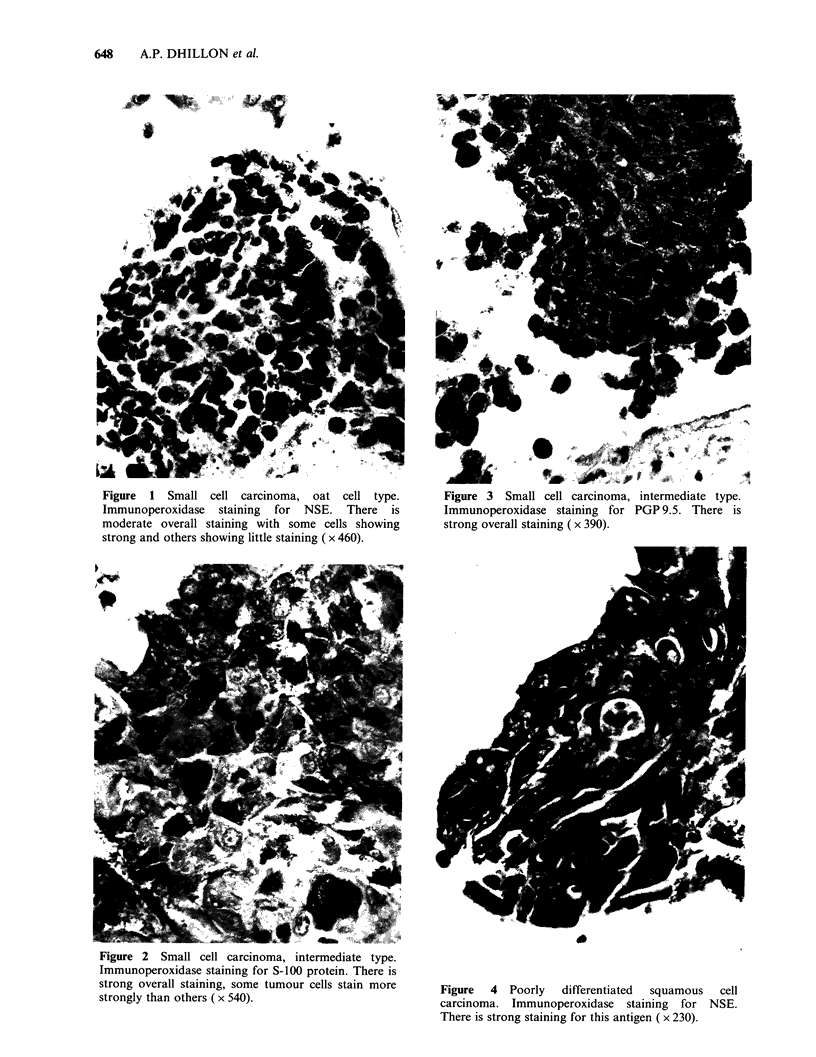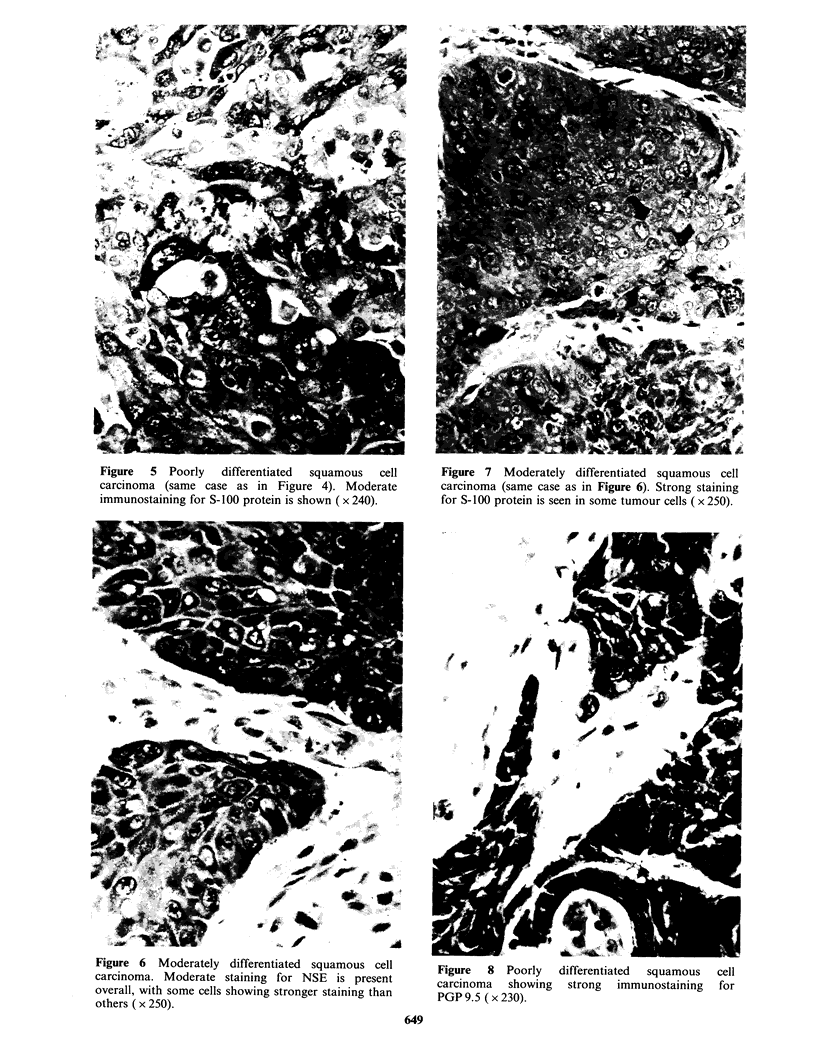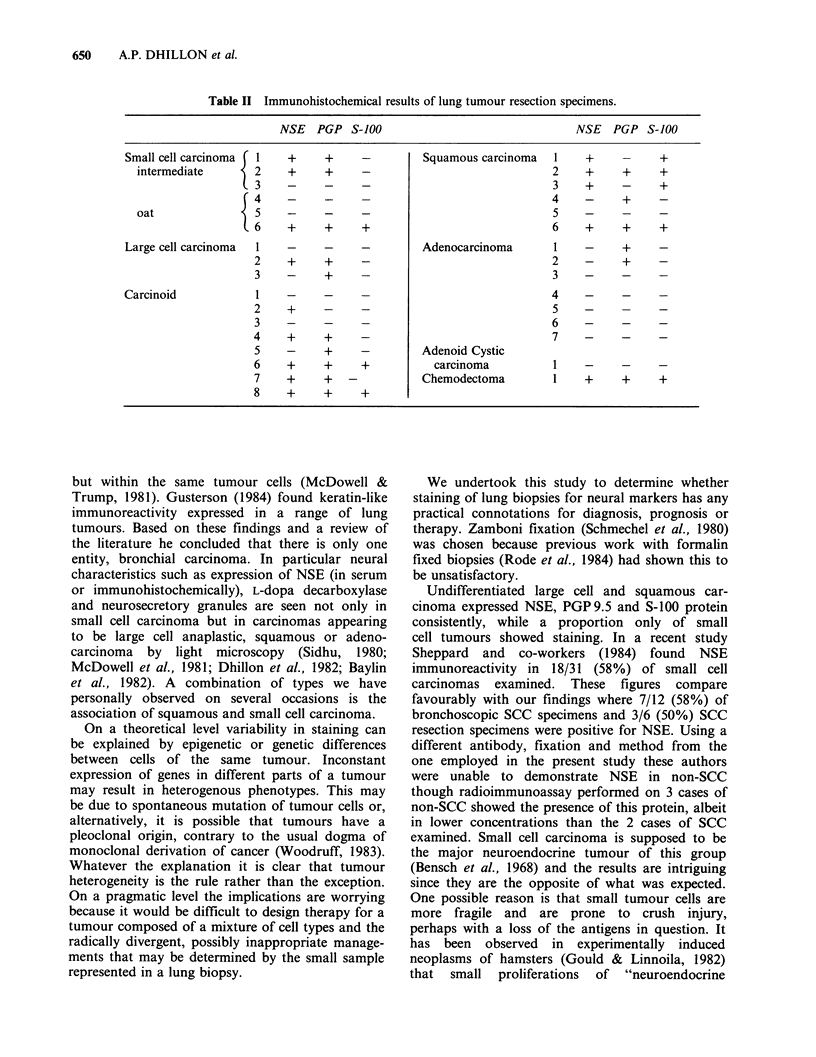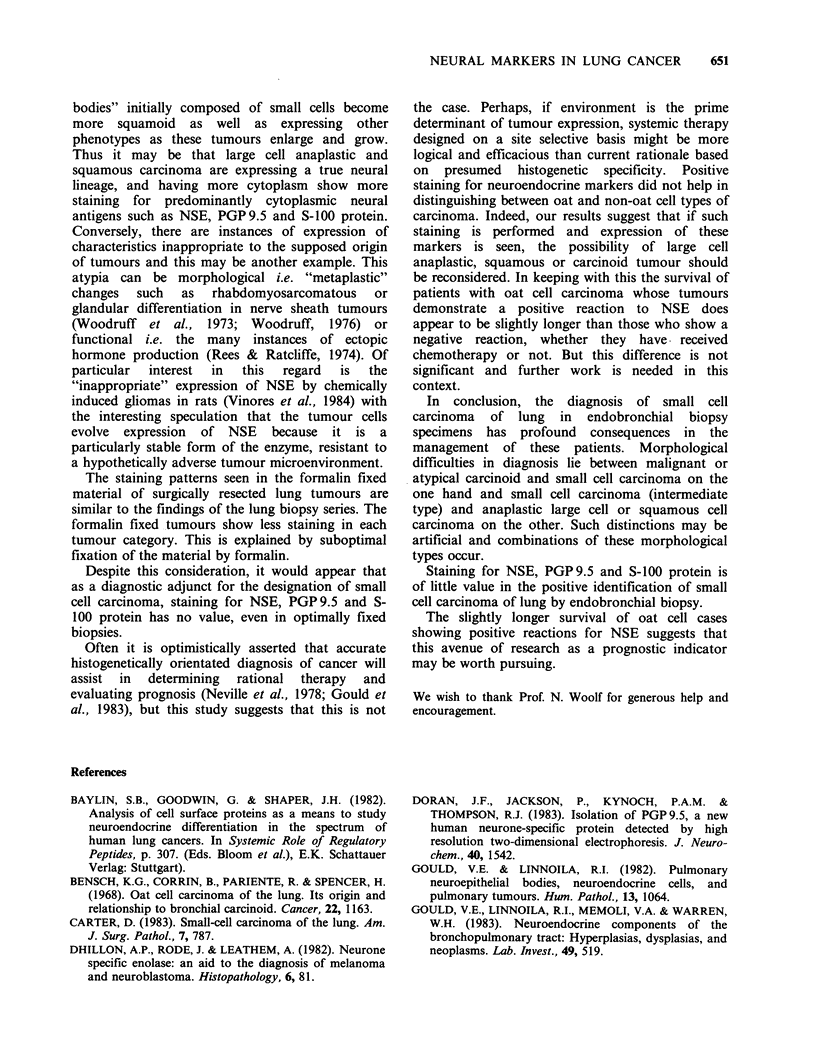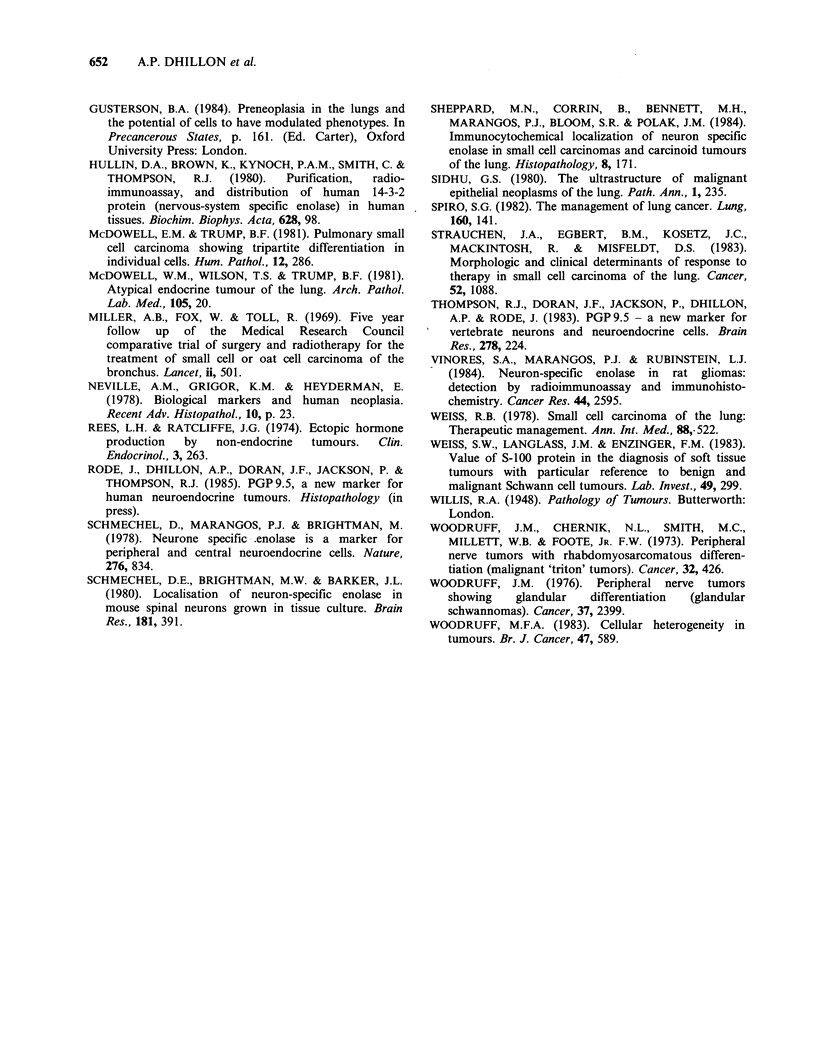# Neural markers in carcinoma of the lung.

**DOI:** 10.1038/bjc.1985.97

**Published:** 1985-05

**Authors:** A. P. Dhillon, J. Rode, D. P. Dhillon, E. Moss, R. J. Thompson, S. G. Spiro, B. Corrin

## Abstract

**Images:**


Br. J. Cancer (1985), 51, 645–652

### Neural markers in carcinoma of the lung

A.P. Dhillon[1], J. Rode[1], D.P. Dhillon[2], E. Moss[1], R.J. Thompson[3], S.G. Spiro[2] & B. Corrin[2]

[1]*The Bland-Sutton Institute of Pathology, The Middlesex Hospital Medical School, London, W1;* [2]*Brompton Hospital, London, SW3 and* [3]*Department of Clinical Biochemistry, School of Clinical Medicine, Addenbrooke's Hospital, Cambridge, UK.*

**Summary** Small cell carcinoma (SCC) is considered to be of neuroendocrine origin. Neurone specific enolase (NSE) and PGP 9.5 are markers of neural and neuroendocrine differentiation. S-100 protein is a marker of glial differentiation. The expression of these markers in endobronchial biopsy and lung tumour resection specimens was studied to see if any diagnostic, prognostic or therapeutic implications would emerge.

Zamboni fixed endobronchial tumour biopsy specimens from 20 patients were examined. Twelve of these were cases of SCC and 8 were non-SCC. Of the 12 SCC, 7 were positive for NSE, 6 for PGP 9.5 and 5 for S-100 protein. Cases which showed a positive reaction for NSE had a mean survival of 9.1 months compared with 3.9 months for those with a negative reaction, but the number of cases is too small to assign any statistical significance. There was no difference in survival times between positive and negative reactors for PGP 9.5 and S-100 protein. All 8 cases of non-SCC showed positive reactions to all three markers.

Of 32 formalin fixed lung tumour resection specimens 6 were cases of SCC, 25 non-SCC and 1 a chemodectoma. Three of the 6 cases of SCC showed positive staining for NSE, 3 for PGP 9.5 and 1 for S-100 protein. Of the 25 non-SCC, 10 were positive for NSE, 12 for PGP 9.5 and 6 for S-100 protein. The 1 chemodectoma stained positively for all three markers.

Neuroendocrine markers are of little value in differentiating SCC from non-SCC. Positive staining for NSE in SCC may be an indicator of prolonged survival but further investigation is required.

The management of lung carcinoma is determined by histological type often based solely upon results of bronchoscopic lung biopsies. A surgical approach constitutes primary treatment for operable cases of squamous carcinoma, adenocarcinoma and carcinoid while such therapy for small cell carcinoma shows poor results (Weiss, 1978; Miller *et al.*, 1969) and therefore radiation and chemotherapy are recommended. Results of these forms of treatment remain poor and median survival, at best, is 11 months (Spiro, 1982).

Small cell carcinoma is considered to be a neuroendocrine type tumour (Bensch *et al.*, 1968; Gould *et al.*, 1983; Carter, 1983). Neurone specific enolase (NSE) and PGP 9.5 are markers of neural and neuroendocrine differentiation (Schmechel *et al.*, 1978; Thompson *et al.*, 1983). S-100 protein is a marker of glial differentiation as well as being found in melanomas, Langerhans cells and cartilagenous tumours (Weiss *et al.*, 1983). We studied the expression of these neural markers in lung biopsy specimens to see if any diagnostic, prognostic or therapeutic implications would emerge, since morphologic assessment alone is inadequate (Strauchen *et al.*, 1983).

Correspondence: J. Rode.
Received 5 July, 1984; and in revised form 30 January 1985.

In addition, a range of resected lung carcinomas were studied to provide a framework for interpretation of immunostaining of the lung biopsies.

### Materials and methods

### *Tissue specimens*

The biopsy study was performed prospectively. Endobronchial biopsies from 20 patients (Table I) were taken as part of the usual assessment of those with lung carcinoma. The tissue was immediately fixed in Zamboni fixative (Schmechel *et al.*, 1980) – a buffered paraformaldehyde, glutaraldehyde and picric acid mixture. Routine paraffin embedding was employed and sections were cut at 5 $\mu$m. One section of each case was stained with haematoxylin and eosin.

An indirect immunoperoxidase technique was applied for the demonstration of NSE, PGP 9.5 and S-100 protein. Endogenous peroxidase was quenched with 3% hydrogen peroxide for 15 min and each of the primary antisera was used at a dilution of 1/100 for 30 min. Swine anti-rabbit globulin conjugated to peroxidase (Dako) was used at 1/50 dilution for 30 min and immunolocalisation was demonstrated with diaminobenzidine with hydrogen peroxide.

© The Macmillan Press Ltd., 1985

**Table I** Clinical data and immunohistochemistry of bronchoscopic specimens.

| | | *Stage* | *Treatment* | *Response* | *Survival* | *NSE* | *PGP* | *S-100* |
|---|---|---|---|---|---|---|---|---|
| SCC | | | | | | | | |
| i | 1 | L | C | CR | >13 mo | + | + | + |
| o | 2 | L | C+D | CR | 18 mo | + | + | + |
| o | 3 | L | C | PR | 10 mo | + | − | − |
| o | 4 | E | C | PR | >15 mo | + | − | − |
| o | 5 | E | C | PR | 10 mo | − | − | − |
| o | 6 | E | C | PR | 4 mo | − | − | + |
| i | 7 | E | C | PR | $1\frac{1}{2}$ mo | + | + | − |
| o | 8 | E | C | PR | $1\frac{1}{2}$ mo | − | + | − |
| o | 9 | E | C | Lost to follow up | | − | − | − |
| o | 10 | E | NT | | 6 mo | + | + | + |
| i | 11 | E | NT | | $\frac{1}{2}$ mo | + | + | + |
| o | 12 | E | NT | | $\frac{1}{4}$ mo | − | − | − |
| NON SCC | | | | | | | | |
| | 1 | L | S | Died post op | | + | + | + |
| | 2 | L | D | − | 1 mo | + | + | + |
| | 3 | E | NT | − | >6 mo | + | + | + |
| | 4 | L | NT | − | >8 mo | + | + | + |
| | 5 | E | NT | − | >11 mo | + | + | + |
| | 6 | E | NT | − | 6 mo | + | + | + |
| | 7 | E | NT | − | $\frac{1}{4}$ mo | + | + | + |
| | 8 | L | S | − | >10 mo | + | + | + |

*KEY:* i = small cell carcinoma, intermediate type; o = small cell carcinoma, oat cell type; L = disease limited to hemithorax (limited); E = disease beyond heimthorax (extensive); C = chemotherapy; D = radiotherapy; S = surgery (pneumonectomy or lobectomy); CR = complete response to chemotherapy as judged by clearing of visible tumour clinically, radiologically and bronchoscopically; PR = partial response to treatment, reduction of tumour but not complete response.

Rabbit anti-NSE and anti-PGP 9.5 were prepared and characterised as described previously (Hullin *et al.*, 1980; Dhillon *et al.*, 1982; Thompson *et al.*, 1983; Doran *et al.*, 1983; Rode *et al.*, 1985). These studies confirmed the specificity of these markers. No staining for NSE and PGP 9.5 of normal non-neural or non-neuroendocrine tissues was observed. The small quantities of NSE and PGP 9.5 found in extracts of tissues usually not regarded as neuroendocrine or neural can be explained by the presence in these tissues of nerves and cell populations representing the diffuse neuroendocrine system. Rabbit anti- S-100 protein was a generous gift from Dako. Negative controls were performed using non-immune serum in place of the specific primary antibody. Absorption studies were not performed. Positive controls comprised a section of nerve which was included with each batch of sections of lung biopsy being immunostained.

Each biopsy was assessed histologically. Immunostaining was assessed initially on a scale of none, slight, moderate and strong staining. Those cases showing none and slight staining only were assigned to a negative category and those showing moderate and strong staining were assigned to a positive category.

The study of lungs resected for carcinoma consisted of sections taken from formalin fixed paraffin processed material taken from the files of the Bland-Sutton Institute of Pathology. Representative sections were cut at 5 $\mu$m and stained (as above) from 6 cases of squamous cell carcinoma (1 well, 3 moderately and 2 poorly differentiated), 7 cases of adenocarcinoma (2 moderately, 4 poorly differentiated and 1 broncho-alveolar cell carcinoma), 8 carcinoid tumours, 3 large cell carcinomas, 6 small cell carcinomas (3 intermediate and 3 oat cell), 1 adenoid cystic carcinoma and 1 chemodectoma (Table II). Metastasis to the lung from other sites was excluded by pre-operative clinical assessment.

### *Clinical assessment*

Clinical assessment included physical examination, radiology, biochemistry, fibreoptic bronchoscopy, iliac crest marrow aspiration and isotope bone scan. Disease extent was judged as being limited or extensive. Limited disease was that confined to one hemithorax (including the ipsilateral supraclavicular fossa) and extensive was outside these limits (Spiro, 1983).

Nine of the 12 patients with small cell carcinoma received specific chemotherapy. This consisted of between 1 and 8 courses of cyclophosphamide, vincristine and VP 16. The bronchoscopy specimen from patient 7 was obtained at relapse following 8 courses of chemotherapy – this patient subsequently received radiotherapy to the primary thoracic site of relapse. Response to chemotherapy was judged to be complete (CR) if there was clearing of visible tumour clinically, radiologically and bronchoscopically; partial response (PR) was anything less than this.

Two of the 8 non-small cell cases underwent surgical resection of the limited tumour. One patient received palliative radiotherapy.

### Results

### *Immunohistochemistry*

Of the 20 endobronchial biopsy specimens there were 12 cases of small cell carcinoma (9 oat cell and 3 intermediate). Two cases of undifferentiated large cell carcinoma, 4 cases of poorly differentiated squamous, 1 moderately differentiated squamous and 1 well differentiated squamous cell carcinoma were represented. The predominance of oat cell tumours in this study reflects the referral of patients with this type of tumour from other centres to one of the authors (S.G.S.) for treatment.

Four small cell carcinomas (2 intermediate, 2 oat cell) (Figures 1–3) and all large cell anaplastic and squamous cell carcinomas (Figures 4–8) stained for NSE, PGP 9.5 and S-100 protein. One small cell carcinoma (intermediate) stained for NSE and PGP 9.5. Four oat cell carcinomas stained for one marker only (2 for NSE, 1 for PGP 9.5 and 1 for S-100 protein). Three oat cell carcinomas did not stain for any of the markers. No staining was seen in the companion sections where non immune serum was substituted for primary antibody. Staining of scattered bronchial epithelial, mucous gland and cartilage cells for each of the markers was seen in occasional biopsies.

In the resection specimens, of 6 squamous carcinomas 4 showed staining for NSE, 3 for PGP 9.5 and 4 for S-100 protein. Of 3 large cell anaplastic carcinomas staining for NSE was present in 1, for PGP 9.5 in 2 and none showed staining for S-100 protein. Six small cell carcinomas were studied, 3 oat and 3 intermediate cell types. Two intermediate type cancers stained for NSE and PGP 9.5, none stained for S-100 protein. Staining of oat cell carcinoma was seen in one case only that was positive for NSE, PGP 9.5 and S-100 protein. No staining for NSE and S100 protein was seen in adenocarcinomas. Two (moderately differentiated) of the 7 adenocarcinomas stained for PGP 9.5. Of the 8 carcinoid tumours, 5 stained for NSE, 5 for PGP 9.5 and 2 for S-100 protein. The 1 chemodectoma represented in the study stained for all 3 markers employed whilst 1 adenoid cystic carcinoma was negative for the 3 antigens.

These results are summarised in the Tables 1 and 2.

### *Clinical correlates*

*Small cell carcinoma* Three of the 12 patients had limited disease and all three received chemotherapy. Two of these 3 showed a complete response and the mean survival of these 3 was 14 months. The 2 complete responders were positive for NSE, PGP 9.5 and S-100 protein; the partial responder was positive only for NSE.

Six of the 9 "extensive" group also received chemotherapy but 1 was lost to follow-up. The remaining 5 showed a partial response to treatment only and their mean survival was 6 months. Two of these 5 were positive for NSE, two for PGP 9.5 and only 1 for S-100 protein.

Three of 12 patients were not treated. Two of these 3 were positive for NSE, PGP 9.5 and S-100 protein; one survived 6 months and the other 2 weeks. The third case was negative for all three markers and survived one week only.

Cases which demonstrated a positive reaction for NSE had a mean survival of $9.1 \pm 7$ months ($n = 7$); those with a negative reaction survived $3.9 \pm 4$ months ($n = 4$). The number of cases is too small to assign any statistical significance.

Comparing positive and negative responders to PGP 9.5 and S-100 protein showed no differences in survival.

*Non-small cell carcinoma* All 8 cases were positive for all 3 neuroendocrine markers. Survival ranged from 1 week to $>11$ months in these patients.

### Discussion

The histogenesis of carcinomas of lung is the subject of current debate. Most tumours can be assigned to a category such as squamous, small cell, adeno or large cell carcinoma on the predominant morphological features. However, with adequate sampling it is recognised that many tumours express a combination of these appearances not only in different parts of the tumour (Willis, 1948)

**Figure 1** Small cell carcinoma, oat cell type. Immunoperoxidase staining for NSE. There is moderate overall staining with some cells showing strong and others showing little staining ( × 460).

**Figure 2** Small cell carcinoma, intermediate type. Immunoperoxidase staining for S-100 protein. There is strong overall staining, some tumour cells stain more strongly than others ( × 540).

**Figure 3** Small cell carcinoma, intermediate type. Immunoperoxidase staining for PGP 9.5. There is strong overall staining ( × 390).

**Figure 4** Poorly differentiated squamous cell carcinoma. Immunoperoxidase staining for NSE. There is strong staining for this antigen ( × 230).

**Figure 5** Poorly differentiated squamous cell carcinoma (same case as in Figure 4). Moderate immunostaining for S-100 protein is shown ( × 240).

**Figure 7** Moderately differentiated squamous cell carcinoma (same case as in **Figure 6**). Strong staining for S-100 protein is seen in some tumour cells ( × 250).

**Figure 6** Moderately differentiated squamous cell carcinoma. Moderate staining for NSE is present overall, with some cells showing stronger staining than others ( × 250).

**Figure 8** Poorly differentiated squamous cell carcinoma showing strong immunostaining for PGP 9.5 ( × 230).

**Table II** Immunohistochemical results of lung tumour resection specimens.

| | | *NSE* | *PGP* | *S-100* | | | *NSE* | *PGP* | *S-100* |
|---|---|---|---|---|---|---|---|---|---|
| Small cell carcinoma intermediate | 1 | + | + | − | Squamous carcinoma | 1 | + | − | + |
| | 2 | + | + | − | | 2 | + | + | + |
| | 3 | − | − | − | | 3 | + | − | + |
| oat | 4 | − | − | − | | 4 | − | + | − |
| | 5 | − | − | − | | 5 | − | − | − |
| | 6 | + | + | + | | 6 | + | + | + |
| Large cell carcinoma | 1 | − | − | − | Adenocarcinoma | 1 | − | + | − |
| | 2 | + | + | − | | 2 | − | + | − |
| | 3 | − | + | − | | 3 | − | − | − |
| Carcinoid | 1 | − | − | − | | 4 | − | − | − |
| | 2 | + | − | − | | 5 | − | − | − |
| | 3 | − | − | − | | 6 | − | − | − |
| | 4 | + | + | − | | 7 | − | − | − |
| | 5 | − | + | − | Adenoid Cystic | | | | |
| | 6 | + | + | + | carcinoma | 1 | − | − | − |
| | 7 | + | + | − | Chemodectoma | 1 | + | + | + |
| | 8 | + | + | + | | | | | |

but within the same tumour cells (McDowell & Trump, 1981). Gusterson (1984) found keratin-like immunoreactivity expressed in a range of lung tumours. Based on these findings and a review of the literature he concluded that there is only one entity, bronchial carcinoma. In particular neural characteristics such as expression of NSE (in serum or immunohistochemically), L-dopa decarboxylase and neurosecretory granules are seen not only in small cell carcinoma but in carcinomas appearing to be large cell anaplastic, squamous or adenocarcinoma by light microscopy (Sidhu, 1980; McDowell *et al.*, 1981; Dhillon *et al.*, 1982; Baylin *et al.*, 1982). A combination of types we have personally observed on several occasions is the association of squamous and small cell carcinoma.

On a theoretical level variability in staining can be explained by epigenetic or genetic differences between cells of the same tumour. Inconstant expression of genes in different parts of a tumour may result in heterogenous phenotypes. This may be due to spontaneous mutation of tumour cells or, alternatively, it is possible that tumours have a pleoclonal origin, contrary to the usual dogma of monoclonal derivation of cancer (Woodruff, 1983). Whatever the explanation it is clear that tumour heterogeneity is the rule rather than the exception. On a pragmatic level the implications are worrying because it would be difficult to design therapy for a tumour composed of a mixture of cell types and the radically divergent, possibly inappropriate managements that may be determined by the small sample represented in a lung biopsy.

We undertook this study to determine whether staining of lung biopsies for neural markers has any practical connotations for diagnosis, prognosis or therapy. Zamboni fixation (Schmechel *et al.*, 1980) was chosen because previous work with formalin fixed biopsies (Rode *et al.*, 1984) had shown this to be unsatisfactory.

Undifferentiated large cell and squamous carcinoma expressed NSE, PGP 9.5 and S-100 protein consistently, while a proportion only of small cell tumours showed staining. In a recent study Sheppard and co-workers (1984) found NSE immunoreactivity in 18/31 (58%) of small cell carcinomas examined. These figures compare favourably with our findings where 7/12 (58%) of bronchoscopic SCC specimens and 3/6 (50%) SCC resection specimens were positive for NSE. Using a different antibody, fixation and method from the one employed in the present study these authors were unable to demonstrate NSE in non-SCC though radioimmunoassay performed on 3 cases of non-SCC showed the presence of this protein, albeit in lower concentrations than the 2 cases of SCC examined. Small cell carcinoma is supposed to be the major neuroendocrine tumour of this group (Bensch *et al.*, 1968) and the results are intriguing since they are the opposite of what was expected. One possible reason is that small tumour cells are more fragile and are prone to crush injury, perhaps with a loss of the antigens in question. It has been observed in experimentally induced neoplasms of hamsters (Gould & Linnoila, 1982) that small proliferations of "neuroendocrine bodies" initially composed of small cells become more squamoid as well as expressing other phenotypes as these tumours enlarge and grow. Thus it may be that large cell anaplastic and squamous carcinoma are expressing a true neural lineage, and having more cytoplasm show more staining for predominantly cytoplasmic neural antigens such as NSE, PGP 9.5 and S-100 protein. Conversely, there are instances of expression of characteristics inappropriate to the supposed origin of tumours and this may be another example. This atypia can be morphological *i.e.* "metaplastic" changes such as rhabdomyosarcomatous or glandular differentiation in nerve sheath tumours (Woodruff *et al.*, 1973; Woodruff, 1976) or functional *i.e.* the many instances of ectopic hormone production (Rees & Ratcliffe, 1974). Of particular interest in this regard is the "inappropriate" expression of NSE by chemically induced gliomas in rats (Vinores *et al.*, 1984) with the interesting speculation that the tumour cells evolve expression of NSE because it is a particularly stable form of the enzyme, resistant to a hypothetically adverse tumour microenvironment.

The staining patterns seen in the formalin fixed material of surgically resected lung tumours are similar to the findings of the lung biopsy series. The formalin fixed tumours show less staining in each tumour category. This is explained by suboptimal fixation of the material by formalin.

Despite this consideration, it would appear that as a diagnostic adjunct for the designation of small cell carcinoma, staining for NSE, PGP 9.5 and S-100 protein has no value, even in optimally fixed biopsies.

Often it is optimistically asserted that accurate histogenetically orientated diagnosis of cancer will assist in determining rational therapy and evaluating prognosis (Neville *et al.*, 1978; Gould *et al.*, 1983), but this study suggests that this is not the case. Perhaps, if environment is the prime determinant of tumour expression, systemic therapy designed on a site selective basis might be more logical and efficacious than current rationale based on presumed histogenetic specificity. Positive staining for neuroendocrine markers did not help in distinguishing between oat and non-oat cell types of carcinoma. Indeed, our results suggest that if such staining is performed and expression of these markers is seen, the possibility of large cell anaplastic, squamous or carcinoid tumour should be reconsidered. In keeping with this the survival of patients with oat cell carcinoma whose tumours demonstrate a positive reaction to NSE does appear to be slightly longer than those who show a negative reaction, whether they have received chemotherapy or not. But this difference is not significant and further work is needed in this context.

In conclusion, the diagnosis of small cell carcinoma of lung in endobronchial biopsy specimens has profound consequences in the management of these patients. Morphological difficulties in diagnosis lie between malignant or atypical carcinoid and small cell carcinoma on the one hand and small cell carcinoma (intermediate type) and anaplastic large cell or squamous cell carcinoma on the other. Such distinctions may be artificial and combinations of these morphological types occur.

Staining for NSE, PGP 9.5 and S-100 protein is of little value in the positive identification of small cell carcinoma of lung by endobronchial biopsy.

The slightly longer survival of oat cell cases showing positive reactions for NSE suggests that this avenue of research as a prognostic indicator may be worth pursuing.

We wish to thank Prof. N. Woolf for generous help and encouragement.

### References

BAYLIN, S.B., GOODWIN, G. & SHAPER, J.H. (1982). Analysis of cell surface proteins as a means to study neuroendocrine differentiation in the spectrum of human lung cancers. In *Systemic Role of Regulatory Peptides*, p. 307. (Eds. Bloom *et al.*), E.K. Schattauer Verlag: Stuttgart).

BENSCH, K.G., CORRIN, B., PARIENTE, R. & SPENCER, H. (1968). Oat cell carcinoma of the lung. Its origin and relationship to bronchial carcinoid. *Cancer*, **22**, 1163.

CARTER, D. (1983). Small-cell carcinoma of the lung. *Am. J. Surg. Pathol.*, **7**, 787.

DHILLON, A.P., RODE, J. & LEATHEM, A. (1982). Neurone specific enolase: an aid to the diagnosis of melanoma and neuroblastoma. *Histopathology*, **6**, 81.

DORAN, J.F., JACKSON, P., KYNOCH, P.A.M. & THOMPSON, R.J. (1983). Isolation of PGP 9.5, a new human neurone-specific protein detected by high resolution two-dimensional electrophoresis. *J. Neurochem.*, **40**, 1542.

GOULD, V.E. & LINNOILA, R.I. (1982). Pulmonary neuroepithelial bodies, neuroendocrine cells, and pulmonary tumours. *Hum. Pathol.*, **13**, 1064.

GOULD, V.E., LINNOILA, R.I., MEMOLI, V.A. & WARREN, W.H. (1983). Neuroendocrine components of the bronchopulmonary tract: Hyperplasias, dysplasias, and neoplasms. *Lab. Invest.*, **49**, 519.

GUSTERSON, B.A. (1984). Preneoplasia in the lungs and the potential of cells to have modulated phenotypes. In *Precancerous States*, p. 161. (Ed. Carter), Oxford University Press: London.

HULLIN, D.A., BROWN, K., KYNOCH, P.A.M., SMITH, C. & THOMPSON, R.J. (1980). Purification, radioimmunoassay, and distribution of human 14-3-2 protein (nervous-system specific enolase) in human tissues. *Biochim. Biophys. Acta*, **628**, 98.

McDOWELL, E.M. & TRUMP, B.F. (1981). Pulmonary small cell carcinoma showing tripartite differentiation in individual cells. *Hum. Pathol.*, **12**, 286.

McDOWELL, W.M., WILSON, T.S. & TRUMP, B.F. (1981). Atypical endocrine tumour of the lung. *Arch. Pathol. Lab. Med.*, **105**, 20.

MILLER, A.B., FOX, W. & TOLL, R. (1969). Five year follow up of the Medical Research Council comparative trial of surgery and radiotherapy for the treatment of small cell or oat cell carcinoma of the bronchus. *Lancet*, **ii**, 501.

NEVILLE, A.M., GRIGOR, K.M. & HEYDERMAN, E. (1978). Biological markers and human neoplasia. *Recent Adv. Histopathol.*, **10**, p. 23.

REES, L.H. & RATCLIFFE, J.G. (1974). Ectopic hormone production by non-endocrine tumours. *Clin. Endocrinol.*, **3**, 263.

RODE, J., DHILLON, A.P., DORAN, J.F., JACKSON, P. & THOMPSON, R.J. (1985). PGP 9.5, a new marker for human neuroendocrine tumours. *Histopathology* (in press).

SCHMECHEL, D., MARANGOS, P.J. & BRIGHTMAN, M. (1978). Neurone specific enolase is a marker for peripheral and central neuroendocrine cells. *Nature*, **276**, 834.

SCHMECHEL, D.E., BRIGHTMAN, M.W. & BARKER, J.L. (1980). Localisation of neuron-specific enolase in mouse spinal neurons grown in tissue culture. *Brain Res.*, **181**, 391.

SHEPPARD, M.N., CORRIN, B., BENNETT, M.H., MARANGOS, P.J., BLOOM, S.R. & POLAK, J.M. (1984). Immunocytochemical localization of neuron specific enolase in small cell carcinomas and carcinoid tumours of the lung. *Histopathology*, **8**, 171.

SIDHU, G.S. (1980). The ultrastructure of malignant epithelial neoplasms of the lung. *Path. Ann.*, **1**, 235.

SPIRO, S.G. (1982). The management of lung cancer. *Lung*, **160**, 141.

STRAUCHEN, J.A., EGBERT, B.M., KOSETZ, J.C., MACKINTOSH, R. & MISFELDT, D.S. (1983). Morphologic and clinical determinants of response to therapy in small cell carcinoma of the lung. *Cancer*, **52**, 1088.

THOMPSON, R.J., DORAN, J.F., JACKSON, P., DHILLON, A.P. & RODE, J. (1983). PGP 9.5 – a new marker for vertebrate neurons and neuroendocrine cells. *Brain Res.*, **278**, 224.

VINORES, S.A., MARANGOS, P.J. & RUBINSTEIN, L.J. (1984). Neuron-specific enolase in rat gliomas: detection by radioimmunoassay and immunohistochemistry. *Cancer Res.* **44**, 2595.

WEISS, R.B. (1978). Small cell carcinoma of the lung: Therapeutic management. *Ann. Int. Med.*, **88**, 522.

WEISS, S.W., LANGLASS, J.M. & ENZINGER, F.M. (1983). Value of S-100 protein in the diagnosis of soft tissue tumours with particular reference to benign and malignant Schwann cell tumours. *Lab. Invest.*, **49**, 299.

WILLIS, R.A. (1948). *Pathology of Tumours*. Butterworth: London.

WOODRUFF, J.M., CHERNIK, N.L., SMITH, M.C., MILLETT, W.B. & FOOTE, JR. F.W. (1973). Peripheral nerve tumors with rhabdomyosarcomatous differentiation (malignant 'triton' tumors). *Cancer*, **32**, 426.

WOODRUFF, J.M. (1976). Peripheral nerve tumors showing glandular differentiation (glandular schwannomas). *Cancer*, **37**, 2399.

WOODRUFF, M.F.A. (1983). Cellular heterogeneity in tumours. *Br. J. Cancer*, **47**, 589.